# Optically Active Oxygen Defects in Titanium Dioxide Doped with Inorganic Acid Ions

**DOI:** 10.3390/nano14121020

**Published:** 2024-06-13

**Authors:** Bin Xu, Xuehui Duan, Tao Zhou, Jinliang Hao, Haotian Qin, Youcai Zhao, Wei Ye, Jianglin Cao

**Affiliations:** 1 State Key Laboratory of Pollution Control and Resource Reuse, School of Environmental Science and Engineering, Tongji University, Shanghai 200092, China; binxu@tongji.edu.cn (B.X.); 2331400@tongji.edu.cn (X.D.); 19614@tongji.edu.cn (T.Z.); jinlianghao@tongji.edu.cn (J.H.); 2310155@tongji.edu.cn (H.Q.); zhaoyoucai@tongji.edu.cn (Y.Z.); 2 Shanghai Institute of Pollution Control and Ecological Security, Shanghai 200092, China; 3 School of Mechanical Engineering, Tongji University, Shanghai 201804, China; weiye@tongji.edu.cn

**Keywords:** TiO_2_, oxygen defect, Ti^3+^, electron transfer, surface modification

## Abstract

Doping inorganic acid ions represents a promising pathway to improving the photocatalytic activity of TiO_2_, and oxygen vacancy has been regarded as the determinant factor for photocatalytic activity. A series of samples doped with Cl^−^, NO_3_^−^, and SO_4_^2−^ was prepared via a simple sol–gel method. Two different oxygen vacancies in the crystal layer of NO_3_^−^/TiO_2_ and Cl^−^/TiO_2_ were found, and those are [Ti^3+^]-V_0_-[Ti^3+^] and [Ti^3+^]-Cl, respectively. The photocurrent of NO_3_^−^/TiO_2_ with [Ti^3+^]-V_0_-[Ti^3+^] is significantly greater than that of Cl^−^/TiO_2_ with [Ti^3+^]-Cl. The least oxygen vacancy is in the gel layer of SO_4_^2−^/TiO_2_, and the negligible photocurrent is due to difficulty in forming a stable sol. Furthermore, the process conditions for the application of TiO_2_ were investigated in this work. The optimal process parameters are to adjust the solution to pH = 3 during sol–gel preparation, to adopt 550 °C as the calcination temperature, and to use an alkaline electrolyte, while the rest of the preparation conditions remain unchanged. This work reveals a new avenue for designing efficient photocatalysts for air pollutant degradation.

## 1. Introduction

In as early as 1911, the term photocatalysis appeared in several research communications [1]. In the photocatalytic materials studied, TiO_2_ has the advantages of high photocatalytic efficiency, stability toward photocorrosion, insolubility in water, low toxicity, and low cost [2,3,4], which makes it a widely used photocatalyst. However, pure TiO_2_ exhibits certain limitations for photocatalytic reactions, such as the wide band gap (∼3.2 eV for brookite and anatase and ∼3.0 eV for rutile) and inefficient charge separation, which somewhat impedes its practical application [5]. To address these shortcomings, doping with inorganic acid ions, notably doping with Cl^−^ and SO_4_^2−^, has emerged as an effective strategy to strongly enhance the photocatalytic activity of TiO_2_. Therefore, it is imperative to investigate the mechanisms underlying the improvement of photocatalytic activity achieved through doping with inorganic acid ions.

R.T. Bento et al. [6] pointed out that the SO_4_^2−^ groups on sulfur-doped photocatalysts stimulated the formation of a highly photoactivated surface. The cationic substitution of the Ti^4+^ by S(VI) resulted in the formation of Ti-O-S bonds, which changed the electronic sites from an O 2p atomic orbital to a S 3p atomic orbital. The oxygen atoms became deficient centers that hindered the recombination rate of the electron (e^−^)/hole (h^+^) pairs under visible light and further increased the formation of •OH radicals. Moreover, Yang et al. [7] pointed out that the adsorption of SO_4_^2−^ on the TiO_2_ surface facilitated the formation of Lewis and Brønsted acid sites, which promoted the adsorption of organics and •OH. Numerous scholars have confirmed that the presence of SO_4_^2−^ promoted the formation of pure anatase [8,9]. Wang [8] firstly proposed a complete process for the formation of pure anatase TiO_2_ in the presence of SO_4_^2−^ based on previous studies [10]. In recent years, Cl^−^-doped TiO_2_ has been extensively reported on. The conventional methods of preparing Cl^−^-doped TiO_2_ions include hydrothermal [11] and sol–gel [12]. Generally speaking, rutile TiO_2_ showed poor photocatalytic activities. However, Badr A. et al. [13] reported that doping Cl^−^ was beneficial for the oxide’s photocatalytic activity by significantly reducing the optical band gap compared with un-doped rutile TiO_2_. Wang et al. [14] prepared a nanorod superstructure rutile Cl^−^-doped TiO_2_, which showed higher photocatalytic activity than commercially available TiO_2_ (P25). In the sol–gel method, the effect of Cl^−^ on the photocatalytic activity of TiO_2_ has also been investigated, but this is controversial. Addamo et al. [15] reported that hydrolyzing isopropyl titanate (TTIP) with the existence of strong acid HCl did not show appreciable photoactivity compared to commercial samplers. The presence of HCl showed detrimental effects on the photocatalytic properties. Conversely, Zhang et al. [16] reported a method for preparing single-phase rutile TiO_2_ nanocrystals by using Ti(OC_4_H_9_)_4_ and HCl at room temperature, which showed greater photocatalytic activity than the commercially available photocatalyst P25. Jung et al. [17] reported that the nanophase titania particles prepared from TTIP by adding HCl at 400 °C and a higher temperature were the rutile/anatase mixed phase, which had higher photoactivity than the pure anatase in the decomposition of trichloroethylene. In addition to SO_4_^2−^ and Cl^−^, the effect of NO_3_^−^ on TiO_2_ was also studied earlier [18,19]. Wang et al. [20] reported an acid-induced assembly strategy for a rutile TiO_2_ photocatalyst on the basis of the treatment of lamellar-protonated titanate with a concentrated HNO_3_ solution. The resulting TiO_2_ product achieved a photocatalytic hydrogen evolution rate of 402.4 μmol h^−1^, which was 3.1 times higher than that of Degussa P25-TiO_2_.

Although the abovementioned studies reported the influence of SO_4_^2−^, Cl^−^, and NO_3_^−^ ions on TiO_2_, the mechanism of these ions on the photocatalytic activity of TiO_2_ is still unclear. Their conclusions were opposite under different experimental conditions. Moreover, few systematic studies have been conducted to compare the effects of the three inorganic acid ions SO_4_^2−^, Cl^−^, and NO_3_^−^, and even fewer studies have focused on the mechanism of their effects, especially nitrate. There are several possible factors affecting photocatalytic activity, such as the crystal phase, surface area, crystal size, and crystallinity of the sample. In this work, the effects of SO_4_^2−^, Cl^−^, and NO_3_^−^ on the crystalline phase, morphology, grain size, and oxygen defects of TiO_2_ are investigated systematically. In addition, the oxygen vacancy structures of SO_4_^2−^-, Cl^−^-, and NO_3_^−^-doped TiO_2_ are proposed in this paper to explain the mechanism of the influence of SO_4_^2−^, Cl^−^, and NO_3_^−^ on TiO_2_ photocatalytic activity.

## 2. Experiments and Methods

### 2.1. Catalyst Preparation

#### 2.1.1. Sol–Gel Preparation

Titanium dioxide (TiO_2_) photocatalysts were synthesized via a sol–gel method. Concentrated acid HCl (38%), HNO_3_ (68%), and H_2_SO_4_ (98%) were used to adjust 150 mL of deionized water to pH = 1, respectively. Tetrabutyl titanate (Ti(OC_4_H_9_)_4_, 12.5 mL) was dissolved into isopropanol (4 mL). The alcohol–ester mixture was added dropwise to the pH = 1 acid solution with rigorous stirring at 80 °C. Then, the mixture was stirred at a constant temperature of 80 °C for 7~8 h. Finally, a certain amount of the anti-polymerization agent PEG (polyethylene glycol 2000) was added. Those milky-white products were denoted as Cl^−^/TiO_2_, NO_3_^−^/TiO_2_, and SO_4_^2−^/TiO_2_, respectively.

#### 2.1.2. Catalyst Powders

The sol was poured into the corundum crucible and placed under the infrared heat lamp until the moisture was dried. It was then calcinated in a muffle furnace at 450 °C for 30 min. The sintered TiO_2_ was ground into a powder and stored in a sealed bag for subsequent material characterization.

#### 2.1.3. Catalyst Electrode

The ITO conductive glass (15~20 Ω) was cut into 2 × 1 cm^2^, ultrasonically cleaned, and washed sufficiently with acetone, concentrated sulfuric acid, and secondary deionized water. It was vertically immersed in the sol and slowly pulled out after staying for 10 s. Then, it was horizontally placed for a while to uniformly distribute the sol on the conductive surface and to be dried with an infrared baking lamp. The above steps were repeated 3 times. The glass substrates with films were placed in a muffle furnace for calcinations at 450 °C for 30 min to form a TiO_2_ electrode. The materials were coated on a conductive glass that appeared to be translucent white after sintering.

### 2.2. Characterization

X-ray diffraction (XRD) patterns were obtained using a D8 Advance X-ray diffractometer with a Ni filter and a graphite monochromator. The X-ray source was Cu Kα radiation with a 2θ diffraction angle ranging from 10 to 80°. The crystallite sizes were calculated according to the Warren and Averbach equations based on the lime spread of the corresponding X-ray diffraction peaks (peaks were fitted using the Voigt function).

(1) $$D=\frac{\lambda \times 180}{\pi \times cos\theta \times L}$$

where *L* is the line width at medium height, λ is the wavelength of the X-ray radiation (0.115406 nm), and θ is the diffracting angle.

X-ray photoelectron spectrometer (XPS) data were recorded on a Kratos AXIS Supra instrument (Kratos Analytical, Manchester, UK) using monochromatic A1 Kα radiation (1486.7 eV, 150 W). Survey scan spectra were acquired using a pass energy of 160 eV and a 1 eV step size. A narrow-region scan was acquired using a pass energy of 40 eV and a 0.1 eV step size. The hybrid lens mode was used in both cases. A charge neutralizer was used throughout the sample-mounting process to electrically isolate the sample from the sample rod. The C1s peak (sp3 peak) of the collected hydrocarbon was spectrally corrected with a binding energy of 285.0 eV. Field emission scanning electron microscopy (SEM) (Model S4800, Hitachi, Tokyo, Japan) was used to obtain the image of the surface morphology. The photocurrent of the photocatalyst was measured in the CHI660D electrochemical workstation (Shanghai Chen Hua Co., Ltd., Shanghai, China) with a three-electrode system by applying a bias voltage of 0.1 V. Conductive glass with a doped TiO_2_ film was the work electrode. The saturated calomel electrode was the reference electrode. The platinum electrode was the counter electrode, and the 0.5 M Na_2_SO_4_ was the electrolyte. The power of the UV Xenon lamp was 80 W. 

## 3. Influence of Preparation Process on Photocurrent

### 3.1. pH of the Electrolyte during Photocurrent Measurement

**Figure 1:**
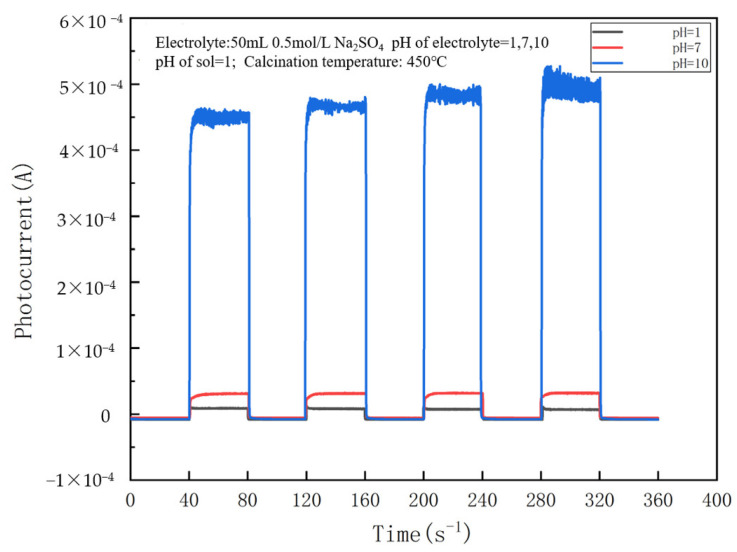
Effect of electrolyte pH on the photocurrent of a NO_3_^−^/TiO_2_ thin-film electrode. (The calcination temperature is 450 °C, and the solution is pH = 1 during sol–gel preparation).

As shown in Figure 1, a higher electrolyte pH leads to a drastic increase in the photocurrent. The photocurrent at electrolyte pH = 10 is about $4.5\times 10^{-4}$ A, which is 15 times the photocurrents at pH = 7, approximately. The charge transfer process that occurs at the TiO_2_/solution interface under illumination actually determines the magnitude of the saturation photocurrent, defining the anodic and cathodic currents across the TiO_2_/solution interface as $I_{a}^{H}$ and $I_{c}^{H}$, respectively. The net photocurrent value can be expressed by the following equation under DC steady-state conditions:

(2) $$I_{net}=I_{a}^{H}-I_{c}^{H}$$

where $I_{a}^{H}$ corresponds to the reaction in which the valence band cavities oxidize OH^−^ or H_2_O occurring at the TiO_2_/solution interface, and $I_{c}^{H}$ corresponds to the reaction in which the conduction band electrons reduce the oxidizing agent in the solution. 

According to the above equation, the net photocurrent depends on the anode current when the cathode current is constant. The anodic current depends on the concentration of OH^−^ ions adsorbed at the Ti atomic sites on the TiO_2_ surface [21]. The higher the concentration of OH^−^ ions, the more excellent the anodic current. When pH > pH_PZC_ (point of zero charge, PZC), the OH^−^ ions adsorbed on the TiO_2_ surface increase by replacing H_2_O molecules. This is favorable for trapping positive protons and improving charge carrier separation. The alkaline condition effectively favors the transfer of photogenerated electrons, which in turn commendably enhances the photocurrent. This well explains the magnitude of photocurrents in the order of pH = 10 ≫ pH = 7 > pH = 1 (Figure 1). A photocurrent is negligible in neutral or acidic electrolytes compared with alkaline conditions.

### 3.2. pH of the Solution during Sol–Gel Preparation

**Figure 2:**
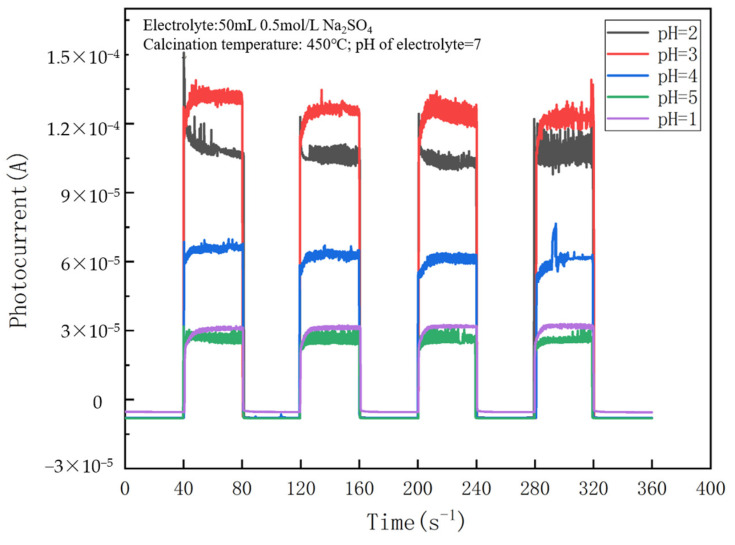
Effect of solution pH during sol–gel preparation on the photocurrent of a NO_3_^−^/TiO_2_ thin-film electrode. (The calcination temperature is 450 °C, and the electrolyte is pH = 7).

The pH of sol–gel effectively influences photocurrents of a thin-film electrode, as shown in Figure 2. The photocurrent is $1.35\times 10^{-4}$ A at pH = 3, which is 4.5 times as high as $3\times 10^{-5}$ A at pH = 1 and 5, approximately. In this experiment, it is clear that the most suitable pH is 3. H^+^ ions under acidic conditions cause Ti (OH)_4_ particles to repel each other, which in turn affects the length of the gelation time. The hydrolysis rate of tetrabutyl titanate is very fast, and whether it can eventually form a stable sol mainly depends on the length of the gelation time. Silva and Kimling gave a detailed explanation of the effect of pH on the gelling time [21,22]. When the pH is <3, the gel time is prolonged with an increase in the pH. The partially free OH^−^ ions of tetrabutyl titanate ([Ti(OH)]^3+^, [Ti(OH)_2_]^2+^, [Ti(OH)_3_]^+^) react with the H^+^ ions in the solution, which generates a relatively stable Ti^4+^ and thus prolongs the gelation time. In contrast, when the pH is >3, the gel time is shortened with an increasing pH. The hydroxide concentration will increase, and the polymerization reaction will be accelerated with the decrease in the solution acidity, and then the gel time is shortened. As discussed in the context of SO_4_^2−^/TiO_2_, it is known that the photocurrent is small if a stable sol cannot be formed successfully.

### 3.3. Mixed Crystal Effect

**Figure 3:**
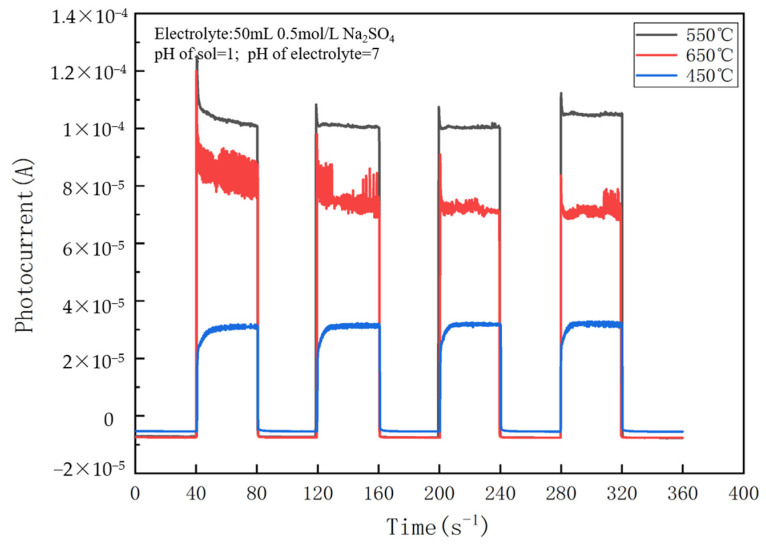
Effect of calcination temperature on the photocurrent of a NO_3_^−^/TiO_2_ thin-film electrode (an electrolyte of pH = 7 and a solution of pH = 1 during sol–gel preparation).

As shown in Figure 3, the photocurrent of the TiO_2_ electrode is the largest when the calcination temperature is 550 °C, which is about $1.0\times 10^{-4}$ A. The photocurrents of TiO_2_ calcined at 550 °C and 650 °C are both much larger than those at 450 °C, which are 2.5~3 times larger. But, when the temperature is >550 °C, the photocurrent is weakened with an increase in the temperature. More impressively, the anatase-type titanium dioxide begins to convert to rutile when the temperature rises to 550 °C, and the proportion of rutile type gradually increases with an increase in the temperature. This property of TiO_2_ can be applied to explain why the magnitude of photocurrents varies with the calcination temperature. This suggests that the TiO_2_ samples contain a certain amount of the rutile phase at 550 °C and 650 °C, which indicates that the existence of a certain proportion of the rutile phase to form mixed crystals can significantly enhance photocurrents compared with the pure anatase type.

**Figure 4:**
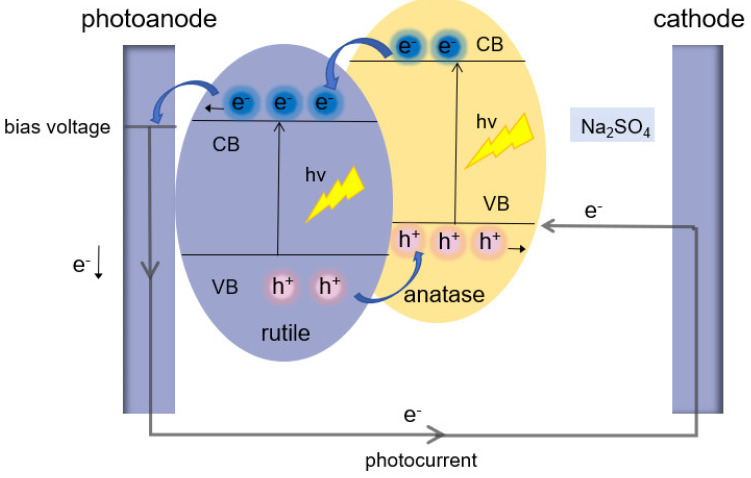
Schematic diagram of photogenerated carrier separation mechanism of a mixed crystal.

As illustrated in Figure 4, the synergistic effect of an anatase–rutile mixed crystal is firstly due to the fact that the conduction band of anatase is more negative than that of rutile. The electrons can be transferred from the anatase conduction band to the rutile conduction band. The energy level barriers between them can inhibit the reserve transfer of electrons, which contributes to the desired separation efficiency of photogenerated carriers. Secondly, the narrower forbidden band width of rutile allows for the extension of the light absorption range of the mixed crystal into the visible region. However, the content of rutile should be appropriate. The rich rutile phase will greatly reduce the probability of electron excitation on the anatase phase, which leads to a tremendous increase in the electron–hole recombination rate. Conversely, when the content of the rutile phase is too limited, the rutile phase particles are covered by a large number of anatase-phase particles. This will inhibit the electron transfer between the anatase and rutile. Therefore, the optimal ratio of rutile and anatase types in a crystal composition is achieved at a calcination temperature of 550 °C. This ratio strikes a balance between the drawbacks associated with a high proportion of the rutile phase at 650 °C and a low proportion at 450 °C.

## 4. Results and Discussion

### 4.1. Sample Morphological Structure and Crystal Phase Type

**Figure 5:**
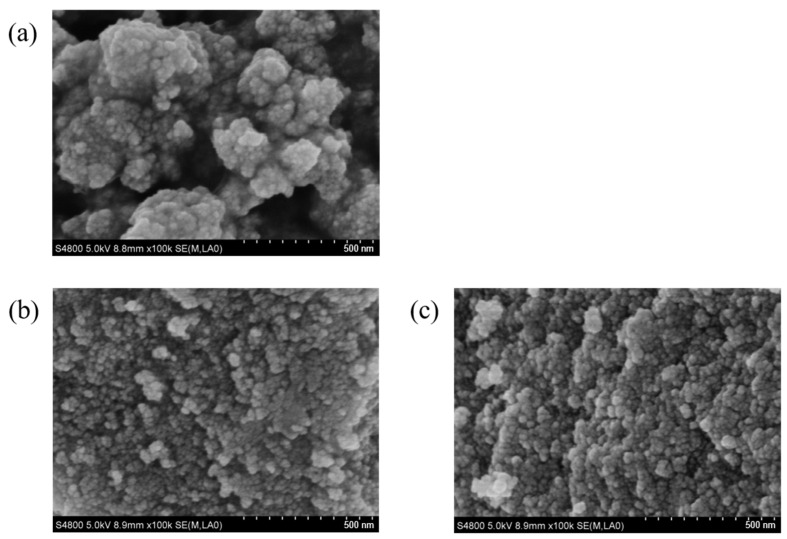
SEM images of SO_4_^2−^/TiO_2_ (**a**), Cl^−^/TiO_2_ (**b**), and NO_3_^−^/TiO_2_ (**c**) at a 10 W magnification.

The morphologies of the SO_4_^2−^/TiO_2_, Cl^−^/TiO_2_, and NO_3_^−^/TiO_2_ samples were revealed by SEM. Almost-spherical anatase particles and clear boundaries between the particles can be observed in three samples, indicating that TiO_2_ particles doped with inorganic acid ions reach a high degree of densification. Figure 5 shows that SO_4_^2−^/TiO_2_ particles exhibit agglomeration, whereas Cl^−^/TiO_2_ and NO_3_^−^/TiO_2_ are uniformly distributed spherical particles, which demonstrates that the type of inorganic acid influences the particle size and the degree of aggregation between particles.

**Figure 6:**
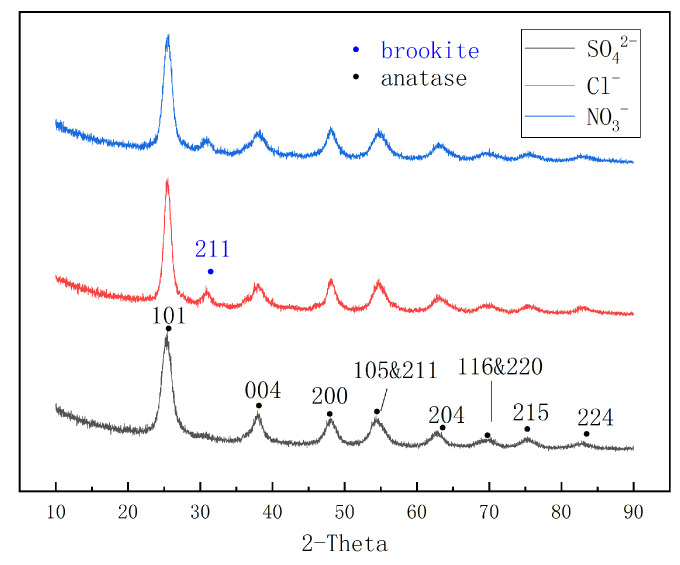
XRD patterns of Cl^−^/TiO_2_, NO_3_^−^/TiO_2_, and SO_4_^2−^/TiO_2._

X-ray diffraction (XRD) measurements were characterized, and the characteristic peaks of the three samples are depicted in Figure 6. Compared with the characterization cards, all diffraction peaks of the series of samples composite within the range of 10°–90° are in accord with monoclinic TiO_2_, showing well-defined peaks corresponding to the architectures of TiO_2_. This indicates that the target substances were successfully synthesized. The strongest diffraction peaks at 2θ = 25.3° are ascribed to the (101) characteristic facets of the anatase phase. Additionally, the diffraction peaks at 2θ = 30.75° are matched with a characteristic crystallographic plane of brookite (211). It can be observed that Cl^−^/TiO_2_ and NO_3_^−^/TiO_2_ both present this characteristic peak, which is due to the fact that it is unattainable to eliminate the brookite phase (minor phase) by adjusting the organic hydrolysis reaction conditions [23]. The low intensity of this peak indicates that the brookite phase accounts for a small proportion of Cl^−^/TiO_2_ and NO_3_^−^/TiO_2_. On the other hand, there is no presence of the brookite phase in SO_4_^2−^/TiO_2_, indicating that the presence of SO_4_^2−^ contributes to the formation of pure anatase TiO_2_. This is consistent with the previous findings of Wang [8] and Kittaka [9].

In addition, the crystal sizes were roughly estimated using the data of XRD and are presented in Table 1. The results are consistent with the observation of SEM. The large grains are formed by the stacking of many small crystals. These results suggest that different acid ions have almost no effect on the crystal size of TiO_2_ but have a significant impact on grain aggregation.

### 4.2. Photocatalytic Current

**Figure 7:**
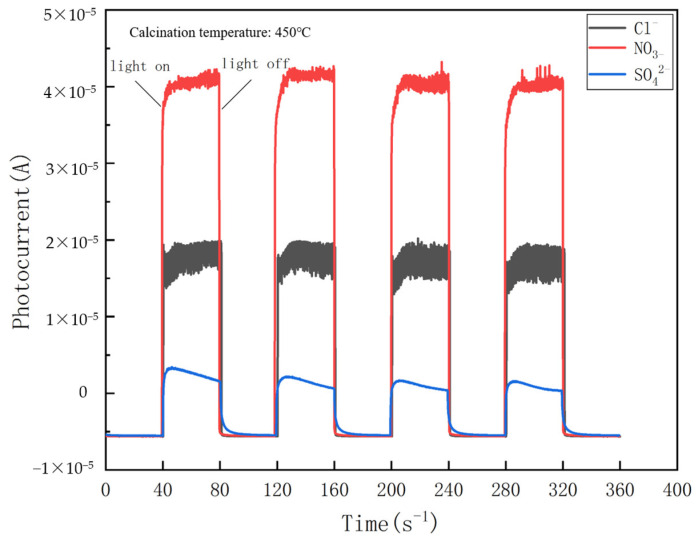
The photocurrents of Cl^−^/TiO_2_, NO_3_^−^/TiO_2_, and SO_4_^2−^/TiO_2_ thin-film electrodes.

Photocurrent measurements were performed for 360 s for the three electrodes, Cl^−^/TiO_2_, NO_3_^−^/TiO_2_, and SO_4_^2−^/TiO_2_, respectively, with the photo current and dark current alternating at 40 s intervals, as shown in Figure 7. Compared with Cl^−^/TiO_2_ and NO_3_^−^/TiO_2_, SO_4_^2−^/TiO_2_ has the smallest photocurrent, which can be generally considered to have almost no photoelectric activity. This could be mainly attributed to the fact that concentrated H_2_SO_4_ mainly exists in the form of sulfuric acid molecules at a low dilution ratio. The insufficient number of H^+^ on the surface of Ti(OH)_4_ particles weakens the mutual repulsive force between the same charges, which leads to an unstable dispersion system and eventually forms precipitates. The precipitate cannot form a transparent and homogeneous TiO_2_ film after impregnation, lifting and sintering on the conductive glass, resulting in poor light transmission. Particle aggregation further impedes the transfer of photogenerated electrons.

The photocurrent of NO_3_^−^/TiO_2_ ($4\times 10^{-5}$ A) is almost as twice as that of Cl^−^/TiO_2_ ($2\times 10^{-5}$ A). The significant difference between the photocurrents of Cl^−^/TiO_2_ and NO_3_^−^/TiO_2_ may be caused by a variety of factors. A preliminary experimental verification revealed that the subtle differences in the surface morphology, crystal phase composition, and crystallite size between Cl^−^/TiO_2_ and NO_3_^−^/TiO_2_ were insufficient to cause a significant discrepancy in the photocurrent. These differences are likely mainly attributed to the Ti^3+^ structure at the surface and/or subsurface oxygen defects.

### 4.3. Oxygen Defects

**Figure 8:**
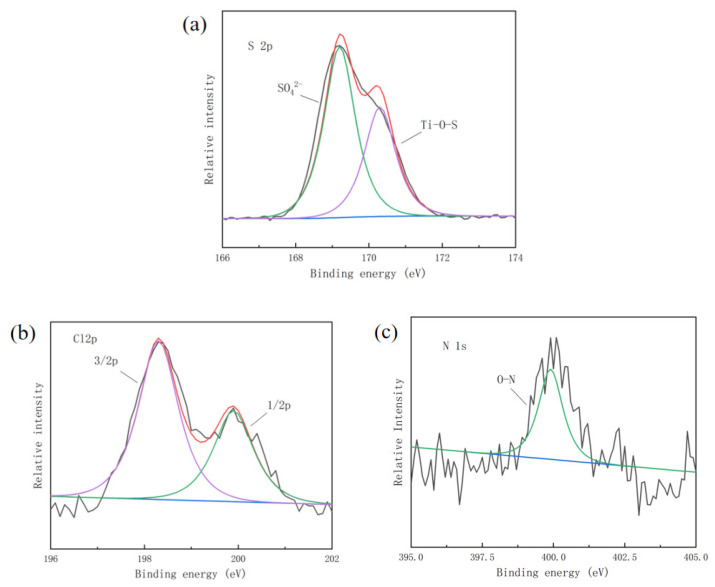
The XPS spectra of SO_4_^2−^/TiO_2_, Cl^−^/TiO_2_, and NO_3_^−^/TiO_2_, with S 2p (**a**); Cl 2p (**b**); and N 1s (**c**); (element S exists mainly in the form of SO_4_^2−^ and Ti-O-S, element Cl exists in the form of Ti-Cl, and element N is attached to Ti in the form of NO_3_^−^).

The chemical species and contents of all elements except for H were analyzed by XPS (Figure 8), and the chemical states of SO_4_^2−^, Cl^−^, and NO_3_^−^ in doped TiO_2_ were also analyzed. As shown in Figure 8a, the peak at 169.2 eV is the strongest peak in the S 2p spectrum, which confirms that the majority of S elements existing in the samples are in the state of SO_4_^2−^. It is observed that two peaks in the Cl 2p spectrum (Figure 8b) at 198.2 eV and 199.8 eV correspond with Cl 2p 3/2 and 2p 1/2 bonded to lattice titanium, respectively. The peak at 198.2 eV is generally considered as evidence for the presence of Ti-Cl bonds, which means that Cl^−^ is absorbed on the TiO_2_ surface. The binding energy peak located at 198.6 eV indicates the presence of Ti-ClO, which suggests that oxygen or an OH radical on the TiO_2_ surface of high heat-treated samples is being used to oxidize adsorbed Cl^−^ into ClO^−^. Guo [24] reported that HCl and HClO were detected in products after a photocatalytic propylene reaction by using Cl^−^-doped TiO_2_ as a catalyst. With a high heat-treated temperature, Cl^−^ ions might be strongly absorbed on the TiO_2_ surface to form a new ClO^−^ species, which affects the Ti^3+^ structure at surface oxygen defects and further causes the difference of photocatalytic currents between Cl^−^/TiO_2_ and NO_3_^−^/TiO_2_. The spectrum of N is shown in Figure 8c. The characteristic peak at 399.9 eV indicates that the N atoms are positioned in the interstitial lattice sites of titanium dioxide rather than substituting for oxygen atoms. Therefore, it is hypothesized that NO_3_^−^ is connected to the lattice titanium through its oxygen atoms, which are usually assumed to be present in the N-O bond. Furthermore, according to the results of a quantitative XPS analysis, the contents of S, Cl, and N in SO_4_^2−^/TiO_2_, Cl^−^/TiO_2_, and NO_3_^−^/TiO_2_ are 3.08%, 0.58%, and 0.35%, respectively. It is shown that the residual amount of elemental N is the lowest among them. This result indicates that most of the NO_3_^−^ ions bonded to the lattice O convert to NO_x_ gas during crystal structure formation, which inhibits the linkage of titanium hydrolysis intermediates and thus retards the crystal growth.

**Figure 9:**
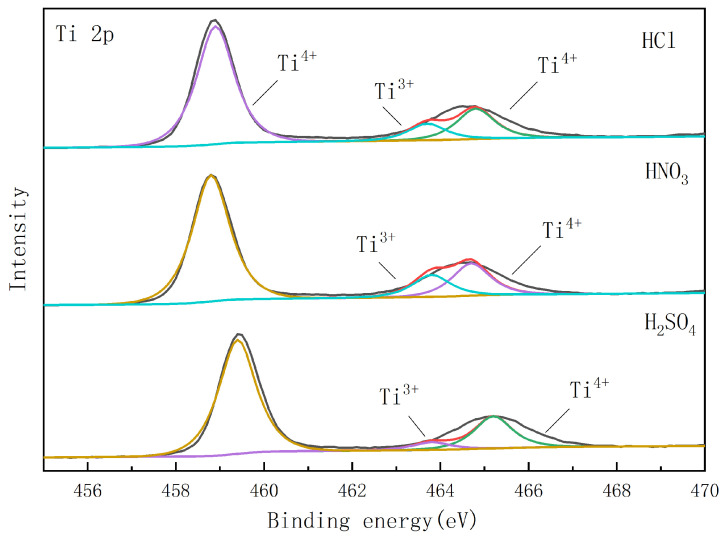
The high-resolution XPS spectra of SO_4_^2−^/TiO_2_, Cl^−^/TiO_2_, and NO_3_^−^/TiO_2_ (Ti 2p). (The calcination temperature is 450 °C, and the solution is pH = 3 during sol preparation).

As shown in Figure 9, the peaks of Ti 2p 3/2 and Ti 2p 1/2 at approximately 459 eV and 465 eV are the major peaks of the three catalysts. The spin–orbit double states splitting (Ti 2p 3/2-Ti 2p 1/2) at about 6 eV indicates that titanium is in the 4+ oxidation state. Meanwhile, it is observed that a small shoulder peak at 463.8 eV is generally considered as the characteristic peak of Ti^3+^ states [25], which are essential for the production of photoelectrons. Compared with Cl^−^/TiO_2_ and NO_3_^−^/TiO_2_, SO_4_^2−^/TiO_2_ contains the least Ti^3+^. The positive displacement of the Ti 2p binding energy can be attributed to the adsorbed SO_4_^2−^ on the TiO_2_ lattice, which reduces the density of the electron cloud around Ti^4+^ and inhibits the formation of a stable sol. However, it is proposed that the significant difference between the photocurrents of Cl^−^/TiO_2_ and NO_3_^−^/TiO_2_ can be explained by the variation in the oxygen defect species and amount of Ti^3+^.

**Figure 10:**
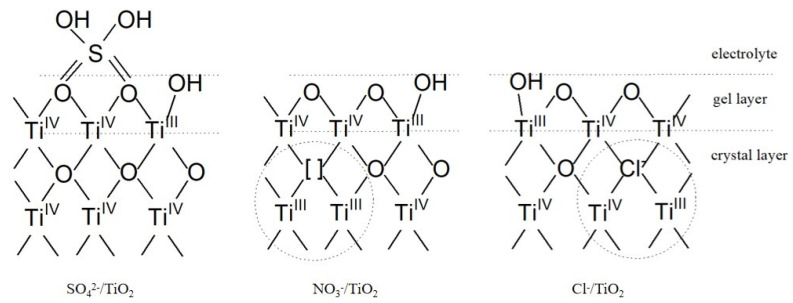
A proposed mechanism of the Ti^3+^-oxygen defect in SO_4_^2−^/TiO_2_, Cl^−^/TiO_2_, and NO_3_^−^/TiO_2_.

According to the principle of electron neutrality, two Ti^3+^ will appear around an oxygen vacancy, and one oxygen vacancy could contribute two free electrons to the Ti 3d conduction band [26]. Payne [27] also confirmed that each vacancy could provide two electrons. An analysis of the spectra of elemental Ti shows that the Ti^3+^ state occurs in the crystal layer of both Cl^−^/TiO_2_ and NO_3_^−^/TiO_2_, as illustrated in Figure 10. During the calcining process of NO_3_^−^/TiO_2_, oxygen vacancies are formed in the crystals after the nitrate ions attached to the titanium atoms are removed as NO_x_ gas. Then, two Ti^3+^ appear in the vicinity of each oxygen vacancy that leads to an oxygen vacancy of [Ti^3+^]-V_0_-[Ti^3+^], and one oxygen vacancy can contribute two free electrons to the conduction band in NO_3_^−^/TiO_2_. However, Cl^−^ ions can partially replace OH groups and adsorb directly to certain defects in Cl^−^/TiO_2_. When the defects containing Cl^−^ are covered with fresh crystals, oxygen vacancies containing Cl^−^ are formed in the TiO_2_ crystals. Under electrically neutral conditions, only one Ti^3+^ state will appear around each oxygen vacancy that leads to an oxygen vacancy of [Ti^3+^]-Cl. One oxygen vacancy can only contribute one free electron to the conduction band of TiO_2_. This further demonstrates that the photocurrent of NO_3_^−^/TiO_2_ is almost twice as that of Cl^−^/TiO_2_.

The abovementioned mechanism explains why the Ti^3+^-oxygen defect causes a difference in photocurrents between Cl^−^/TiO_2_ and NO_3_^−^/TiO_2_. The effect of the Ti^3+^-oxygen defect on the photocatalytic activity is manifested in two aspects: (1) forming a doping energy level below the conduction band, which reduces the excitation energy of electrons, and (2) acting as the active point site of the photocatalytic reaction and the electron capture center, which accelerates the transfer of the electrons and thus improves the separation efficiency. These are all beneficial to overcome the disadvantages of the narrow light absorption range and the high photogenerated carrier recombination rate of anatase titanium dioxide. Theoretically, Ti^3+^ and oxygen vacancies need to coexist to stabilize surface and/or subsurface defects. The oxygen vacancies, as specific sites, can improve the adsorption of O_2_ molecules. This will further produce more superoxide reactive radicals (O_2_•^−^) involved in the degradation reaction process of organic matter. Meanwhile, oxygen vacancies can also trap photogenerated electrons, which facilitates the separation of photogenerated carriers. Additionally, Ti^3+^ can accelerate the transfer of photogenerated electrons and improve the conductivity of TiO_2_. The photogenerated electrons are rapidly transferred to the Ti^3+^ center after being excited and then to the conduction band of TiO_2_. Because the electrons in the Ti^3+^-doped energy level are more easily excited into photogenerated carriers, the energy required for the transfer of electrons from the doped energy level to the conduction band of TiO_2_ is much smaller than that from the valence band to the conduction band. Electron transport channels are formed inside TiO_2_, which can greatly improve the separation efficiency between photogenerated electrons and holes. Furthermore, the existence of Ti^3+^ leads to a drastic increase in surface-bridged OH groups. The high concentration of OH groups in the surface area react with photogenerated cavities to produce •OH, which can also actively participate in the oxidation of organic matter [28]. Therefore, the structure of the Ti^3+^-oxygen defect can strongly enhance photocatalytic activity.

## 5. Conclusions

In short, the photocurrent of Cl^−^/TiO_2_ is significantly reduced compared to that of NO_3_^−^/TiO_2_, and the XPS spectra results demonstrate that the discrepancy in the type of oxygen defects is the crucial reason. Two different oxygen vacancies in the crystal layer of NO_3_^−^/TiO_2_ and Cl^−^/TiO_2_ were found, and those are [Ti^3+^]-V_0_-[Ti^3+^] and [Ti^3+^]-Cl, respectively. There is no oxygen vacancy in the crystal layer of SO_4_^2−^/TiO_2_, and the negligible photocurrent is due to difficulty in forming a stable sol. In summary, the maximum photocurrent observed in the NO_3_^−^/TiO_2_ photocatalyst is attributed to the presence of more Ti^3+^. Thus, in practical photocatalytic applications, nitric acid can be selected as the most suitable pH adjuster to prepare titanium dioxide nanoparticles. Additionally, this study identified optimal process conditions, i.e., adjusting the solution to pH = 3 during sol–gel preparation, adopting 550 °C as the calcination temperature, and using an alkaline electrolyte, while keeping the rest of the preparation conditions constant. This set of process parameters provides a new strategy for the effective utilization of TiO_2_ electrodes in practical pollutant-treatment applications.

## Figures and Tables

**Table 1:** Crystal sizes of SO_4_^2−^/TiO_2_, Cl^−^/TiO_2_, and NO_3_^−^/TiO_2._

Material	Crystal Sizes (nm)
SO_4_^2−^/TiO_2_	4.74
Cl^−^/TiO_2_	5.03
NO_3_^−^/TiO_2_	4.47

## Data Availability

The raw data supporting the conclusions of this article will be made available by the authors on request.

## References

[1] Eibner A. (1911). Action of light on pigments I. Chem.-Ztg..

[2] Fujishima A., Honda K. (1972). Electrochemical photolysis of water at a semiconductor electrode. Nature.

[3] Barraud E., Bosc F., Edwards D., Keller N., Keller V. (2005). Gas phase photocatalytic removal of toluene effluents on sulfated titania. J. Catal..

[4] Yu J., Zhou M., Cheng B., Zhao X. (2006). Preparation, characterization and photocatalytic activity of in situ N, S-codoped TiO_2_ powders. J. Mol. Catal. A-Chem..

[5] Huang H., Zhang T., Cai X., Guo Z., Fan S., Zhang Y., Lin C., Gan T., Hu H., Huang Z. (2021). In situ one-pot synthesis of C-decorated and Cl-doped sea-urchinlike rutile titanium dioxide with highly efficient visible-light photocatalytic activity. ACS Appl. Mater. Interfaces.

[6] Bento R.T., Correa O.V., Antunes R.A., Pillis M.F. (2021). Surface properties enhancement by sulfur-doping TiO_2_ films. Mater. Res. Bull..

[7] Yang R., Li W., Chen N. (1998). Reversible Chemisorption of Nitric Oxide in the Presence of Oxygen on Titania and Titania Modified with Surface Sulfate. Appl. Catal. A Gen..

[8] Wang W., Liu Y., Xue T., Li J., Chen D., Tao Q. (2015). Mechanism and Kinetics of Titanium Hydrolysis in Concentrated Titanyl Sulfate Solution Based on Infrared and Raman Spectra. Chem. Eng. Sci..

[9] Kittaka S., Matsuno K., Takahara S. (1997). Transformation of Ultrafine Titanium Dioxide Particles from Rutile to Anatase at Negatively Charged Colloid Surfaces. J. Solid State Chem..

[10] Parra R., Goes M.S., Castro M.S., Longo E., Bueno P.R., Varela J.A. (2008). Reaction Pathway to the Synthesis of Anatase Via the Chemical Modification of Titanium Isopropoxide with Acetic Acid. Chem. Mater..

[11] Cheng H., Ma J., Zhao Z., Qi L. (1995). Hydrothermal preparation of uniform nano-size rutile and anatase particles. Chem. Mat..

[12] Yamazaki S., Ichikawa K., Saeki A., Tanimura T., Adachi K. (2010). Photocatalytic Degradation of Chlorinated Ethanes in the Gas Phase on the Porous TiO_2_ Pellets: Effect of Surface Acidity. J. Phys. Chem. A.

[13] Badr A., Abdelfettah B. First-principles DFT investigation of the photocatalytic capability of Cl doped rutile TiO_2_ as a self-cleaning coating for photovoltaic panels. Proceedings of the 2021 9th International Renewable and Sustainable Energy Conference (IRSEC).

[14] Wang Y., Zhang L., Deng K., Chen X., Zou Z. (2007). Low temperature synthesis and photocatalytic activity of rutile TiO_2_ nanorod superstructures. J. Phys. Chem. C.

[15] Addamo M., Augugliaro V., Di Paola A., Garcia-Lopez E., Loddo V., Marci G., Molinari R., Palmisano L., Schiavello M. (2004). Preparation, characterization, and photoactivity of polycrystalline nanostructured TiO_2_ catalysts. J. Phys. Chem. B.

[16] Zhang S., Liu C., Liu Y., Zhang Z., Mao L. (2009). Room temperature synthesis of nearly monodisperse rodlike rutile TiO_2_ nanocrystals. Mater. Lett..

[17] Jung K., Park S.B. (1999). Anatase-phase titania: Preparation by embedding silica and photocatalytic activity for the decomposition of trichloroethylene. J. Photochem. Photobiol. A-Chem..

[18] Bacsa R.R., Gratzel M. (1996). Rutile Formation in Hydrothermally Crystallized Nanosized Titania. J. Am. Ceram. Soc..

[19] Bischoff B.L., Anderson M.A. (1995). Peptization Process in the Sol-Gel Preparation of Porous Anatase TiO_2_. Chem. Mater..

[20] Wang H., Hu X., Ma Y., Zhu D., Li T., Wang J. (2020). Nitrate-groupgrafting-induced assembly of rutile TiO_2_ nano-bundles for enhanced photocatalytic hydrogen evolution. Chin. J. Catal..

[21] Silva C.G., Faria J.L. (2009). Effect of Key Operational Parameters on the Photocatalytic Oxidation of Phenol by Nanocrystalline Sol-Gel TiO_2_ under UV Irradiation. J. Mol. Catal. A Chem..

[22] Kimling M.C., Caruso R.A. (2012). Sol-Gel Synthesis of Hierarchically Porous TiO_2_ Beads Using Calcium Alginate Beads as Sacrificial Templates. J. Mater. Chem..

[23] Wang C., Ying J. (1999). Sol-gel synthesis and hydrothermal processing of anatase and rutile titania nanocrystals. Chem. Mat..

[24] Guo J., Mao L., Zhang J., Feng C. (2010). Role of Cl^−^ ions in photooxidation of propylene on TiO_2_ surface. Appl. Surf. Sci..

[25] He Z., Cai Q., Wu M., Shi Y., Fang H., Li L., Chen J., Chen J., Song S. (2013). Photocatalytic Reduction of Cr (VI) in an Aqueous Suspension of Surface-Fluorinated Anatase TiO_2_ Nanosheets with Exposed {001} Facets. Ind. Eng. Chem. Res..

[26] Cao J., Zhao H., Cao F., Zhang J. (2007). The influence of F^−^ doping on the activity of PbO_2_ film electrodes in oxygen evolution reaction. Electrochim. Acta.

[27] Payne D.J., Egdell R.G., Hao W., Foord J.S., Walsh A., Watson G.W. (2005). Why is lead dioxide metallic?. Chem. Phys. Lett..

[28] Du J., Wu Q., Zhong S., Gu X., Liu J., Guo H., Zhang W., Peng H., Zou J. (2015). Effect of Hydroxyl Groups on Hydrophilic and Photocatalytic Activities of Rare Earth Doped Titanium Dioxide Thin Films. J. Rare Earths.

